# Venomous snakebites: Rapid action saves lives—A multifaceted community education programme increases awareness about snakes and snakebites among the rural population of Tamil Nadu, India

**DOI:** 10.1371/journal.pntd.0008911

**Published:** 2020-12-31

**Authors:** Stephen Paul Samuel, Soundararaj Chinnaraju, Harry F. Williams, Elamaran Pichamuthu, Mangaiyarkkarasai Subharao, Mohanraj Vaiyapuri, Sundhararajan Arumugam, Rajendran Vaiyapuri, M. Fazil Baksh, Ketan Patel, Steven A. Trim, Tracey E. Duncombe, Sakthivel Vaiyapuri

**Affiliations:** 1 Queen Elizabeth Hospital, Kings Lynn, United Kingdom; 2 TCR Multispeciality Hospital, Krishnagiri, Tamil Nadu, India; 3 Toxiven Biotech Private Limited, Coimbatore, Tamil Nadu, India; 4 Trichy SRM Medical College Hospital & Research Centre, Tamil Nadu, India; 5 Department of Mathematics and Statistics, University of Reading, Reading, United Kingdom; 6 School of Biological Sciences, University of Reading, Reading, United Kingdom; 7 Venomtech Limited, Sandwich, United Kingdom; 8 Research and Enterprise Services, University of Reading, Reading, United Kingdom; 9 School of Pharmacy, University of Reading, Reading, United Kingdom; Universidad de Costa Rica, COSTA RICA

## Abstract

The lack of public awareness surrounding the dangers of snakebite envenomation (SBE) is one of the most critical factors contributing to SBE-induced complications, and subsequently exacerbating the number of deaths and disabilities resulting from SBE. In this study, we deployed a multifaceted community education programme to educate students, healthcare professionals and members of the public in rural areas of Tamil Nadu, India about the dangers of SBE, appropriate first aid measures and the ‘do’s and don’ts’ following a snakebite. An assessment of prior knowledge within these communities identified several misconceptions concerning snakes and SBE. Using a combination of direct engagement (estimated to reach over 200,000 people), information leaflets (200,000 distributed), posters, video documentaries, media and social media (>2.8 million engagements), over the course of one year (January to December 2019) we reached over 3 million people in rural Tamil Nadu (around 8% of population). Evaluation of community-based assemblies indicated that at least 90% of attendees were able to recall the key messages at the end of the events, and at least 85% were able to recall the key messages even after 12 months. Due to high demand, a one-day symposium was organised to provide clinical knowledge and training on SBE to 250 healthcare professionals in rural Tamil Nadu. Notably, an assessment of patient data (291 victims) collected from a snakebite referral hospital over the same 12-month period (2019) indicated that arrival time at hospital following a snakebite was significantly faster and the effective first aid measures were administered to patients who were aware of our activities compared to those that were not. Overall, our approach provides a framework on how to educate rural communities about the dangers of SBE and thereby, mitigate delayed SBE treatment leading to an overall reduction in SBE-induced mortality, morbidity, treatment costs and other socio-economic ramifications.

## Author summary

Snakebite envenomation (SBE) is a major neglected tropical disease that primarily affects poor communities living in rural areas of developing countries. The lack of knowledge about snakes and SBE is a major factor in augmenting SBE-induced disabilities and deaths. This study reports the significance of a multidimensional community education approach for the dissemination of public health information on SBE across the state of Tamil Nadu in Southern India. We also highlight the extent to which misconceptions about snakes and SBE treatment are held by people in rural communities of Tamil Nadu and demonstrate that key messages and appropriate actions can be effectively communicated through targeted engagement activities and use of the mass media. A one-day symposium provided clinical knowledge and training to healthcare professionals in rural areas about SBE. By providing appropriate awareness about snakes and SBE among the rural population through a variety of approaches, we should be able to reduce SBE-induced complications and the resulting disabilities and deaths. The approaches used in this study can be used as a proof of concept to educate rural communities in other parts of India and also other countries in the world where SBE is a significant issue.

## Introduction

Snakebite envenomation (SBE) is an occupational health hazard that predominantly affects poor communities and has been classified as a high priority neglected tropical disease by the World Health Organisation [1–3]. SBE kills an estimated 140,000 people and causes approximately 500,000 amputations and other forms of permanent disabilities each year. SBE primarily affects rural populations, particularly those living in developing countries [2,4]. Notably, India has been dubbed the “snakebite capital of the world” by some media outlets [5], due to the large number of deaths and disabilities occurring there as a result of SBE. According to the Million Death Study, around 46,000 deaths occur in India alone every year due to SBE [6] and their recent study reports a higher mortality estimate of around 58,000 deaths [7]. Over 200 species of snakes have been identified in India and around 60 of these are venomous. However, the ‘Big Four’ snakes [Russell’s viper (*Daboia russelii)*, Indian cobra (*Naja naja*), Indian krait (*Bungarus caeruleus*) and saw-scaled viper (*Echis carinatus*)] are responsible for the vast majority of deaths and disabilities in this country [6,8–10]. At the state level, Tamil Nadu is one of the most seriously afflicted states by SBE [6–8]. It is also one of the most highly populated Indian states with a population of around 72 million, of which over half (37 million people) live in rural areas with the majority reliant on agriculture-based occupations for their livelihoods [11].

Based on a household survey conducted amongst the rural population of Tamil Nadu, we have previously reported [8] that the incidence of SBE could be up to 3.5 times higher than formerly (around 3,000 every year [6]) estimated. Our estimates indicate that as many as 10,000 people are likely to die every year from SBE in the rural population of Tamil Nadu, and this number is likely to increase significantly in years with a higher annual rain fall. Through interviews with SBE victims and their relatives, we have reported the high socio-economic burden of SBE on rural families [8]. Among the contributing factors, lack of awareness of snakes and SBE was identified as the most critical factor in exacerbating SBE-induced complications, deaths, disabilities and/or increased treatment costs [8,12,13]. Although the population as a whole is aware of the danger of snakes and snakebites (including venomous and dry bites), the majority of people, including some healthcare professionals, were not aware of how to respond to snakes or snakebites. It was also evident from our previous survey that inappropriate first aid practices, such as applying tourniquets, incisions, and sucking blood were common in rural areas [8]. All these activities delay hospital treatment and exacerbate SBE-induced complications which consequently become more of a challenge to treat in hospital settings [12]. Among various reasons, the treatment seeking behaviour of victims or their relatives is largely influenced by traditional myths surrounding snakes and snakebites, and the pressure to seek cheaper, more accessible, traditional treatment which unfortunately delays essential hospital treatment (i.e. they seek hospital treatment only when the situation has become an emergency) [14,15]. The treatment costs in private hospitals are another major concern, although free SBE treatment is available in government hospitals.

Despite reports emphasising the impact of SBE on the rural population of Tamil Nadu [6,8], no significant attempts have been made across the state to improve public education and awareness of SBE. Hence, SBE and its socio-economic ramifications are continuing to alter the lives of thousands of rural people every year [8]. Here, we have designed and implemented a multifaceted programme to educate the rural population of Tamil Nadu about the dangers of snakes and SBE. The aim of this programme was to dispel myths, providing clear advice on how to react to snakes and SBE. Ultimately, the objective was to change the treatment seeking behaviour of SBE victims and their families, for example by reducing the prevalence of traditional remedies, increasing the uptake of effective first aid measures, and prompt arrival at hospital. Our ‘*Venomous snakebites*: *rapid action saves lives’* campaign has reached over 3 million people in rural Tamil Nadu, and it demonstrates the changes in essential knowledge about SBE and treatment seeking behaviour among the rural population of Tamil Nadu. We strongly believe that this approach can be used as a framework for other community education initiatives among rural populations of the world where SBE is a major concern.

## Materials and methods

### Ethical statement

The SBE patients’ data collection, information sheet and consent forms were approved by the Institutional Ethics Committee at ToxiVen Biotech Private Limited, Tamil Nadu, India. Written informed consent was obtained from all the victims prior to data collection. Where the victims were minor (under 18 years old), the written consents were obtained from their parents or legal guardians. The use of pictorial questionnaire was exempted from the Institutional Ethics Committee as it did not collect any personal identifying information from the study participants. All the data collected in this study were analysed anonymously.

### Development of the campaign

The core public health message that we wanted to convey was that rapid action saves lives. This was adopted as the title of our campaign, i.e. ‘*Venomous snakebites*: *rapid action saves lives*’. Specific key messages were subsequently developed in collaboration with researchers in the snakebite field; clinicians who treat snakebite victims and public engagement professionals. These are; (1) stay calm following a bite and move away slowly from the snake to avoid another bite, (2) do not attempt to attack or kill the snake, (3) call for help or walk to get help if necessary, (4) avoid running as this may increase the rate at which the venom spreads through the body, (5) remove any jewellery or tight-fitting clothes from the bitten limb as it may start to swell, (6) do not tie a rope or cloth above the bite site, (7) do not cut and bleed or suck the bite site, (8) get to a hospital as quickly as possible and (9) do not waste time on traditional remedies because they are ineffective. These key messages formed the basis of our community education materials and activities, and subsequent evaluation.

### Study design

This public education programme builds on our previous household survey and socio-economic study which demonstrated a clear need to raise awareness of SBE in Tamil Nadu. Building on the authors’ familiarity with the state and existing network of contacts, this study was designed specifically for Tamil Nadu in South India. The activities were targeted at rural communities with the aim to reach at least 10% (around 3.7 million) of the total rural population. Tamil Nadu has 37 revenue districts, of which, Chennai (a major metropolitan city and the capital of the state) and The Nilgiris (a hill station) were not included in this study due to the lower incidence of snakebites in these areas. For the remaining 35 districts, we aimed to visit at least 2–3 villages in each district, and between 2–4 educational institutions in proximity to these villages. Through direct public engagement activities, we aimed to reach around 0.5% (i.e. around 185,000) of the rural population and to further increase our reach via media and social media to another 9.5% of the rural population. The study locations were selected to cover a wide range of geographical regions (such as coastal, terrestrial, forest, dry and humid areas) across the state where agriculture is the predominant occupation. The programme was developed to be performed in educational institutions and public places in rural villages such as temples, bus/train stations, markets and community centres to reach a cross section of society. We also enlisted the support of educational institutions, non-governmental organisations (NGOs), hospitals, pharmacies and other public places to distribute the information leaflets and, in some cases, to perform additional programme activities. The data provided in this report were obtained between 1^st^ January 2019 and 31^st^ December 2019 although the activities of this project are continuing in Tamil Nadu through our established contact network.

### Tools generated for community education

#### SBE information video

Previous research suggests that information videos are an effective way of teaching lay people about healthcare issues [16,17]. Hence, we developed a 20-minute-long video which played a central role in our community-based engagement with educational establishments and villages in rural Tamil Nadu. The video, produced in Tamil, was directed by a journalist and recorded/edited by professional film makers. It included interviews with a snakebite scientist (SV), two clinicians (SPS & SC) who treat SBE victims regularly, two snake rescuers and members of the public including some SBE victims who had recently been treated in Tamil Nadu. The video covered general information about SBE in general, the ‘Big Four’ snakes, and signs and symptoms of their bites, do’s and don’ts following a snakebite, how to avoid snakebites, how to prevent snakes coming into households and the current treatment methods available for SBE. The video was rigorously reviewed by film makers, public engagement professionals and clinicians and edited as necessary prior to using it in the campaign.

#### SBE Information leaflet and posters

**Fig 1:**
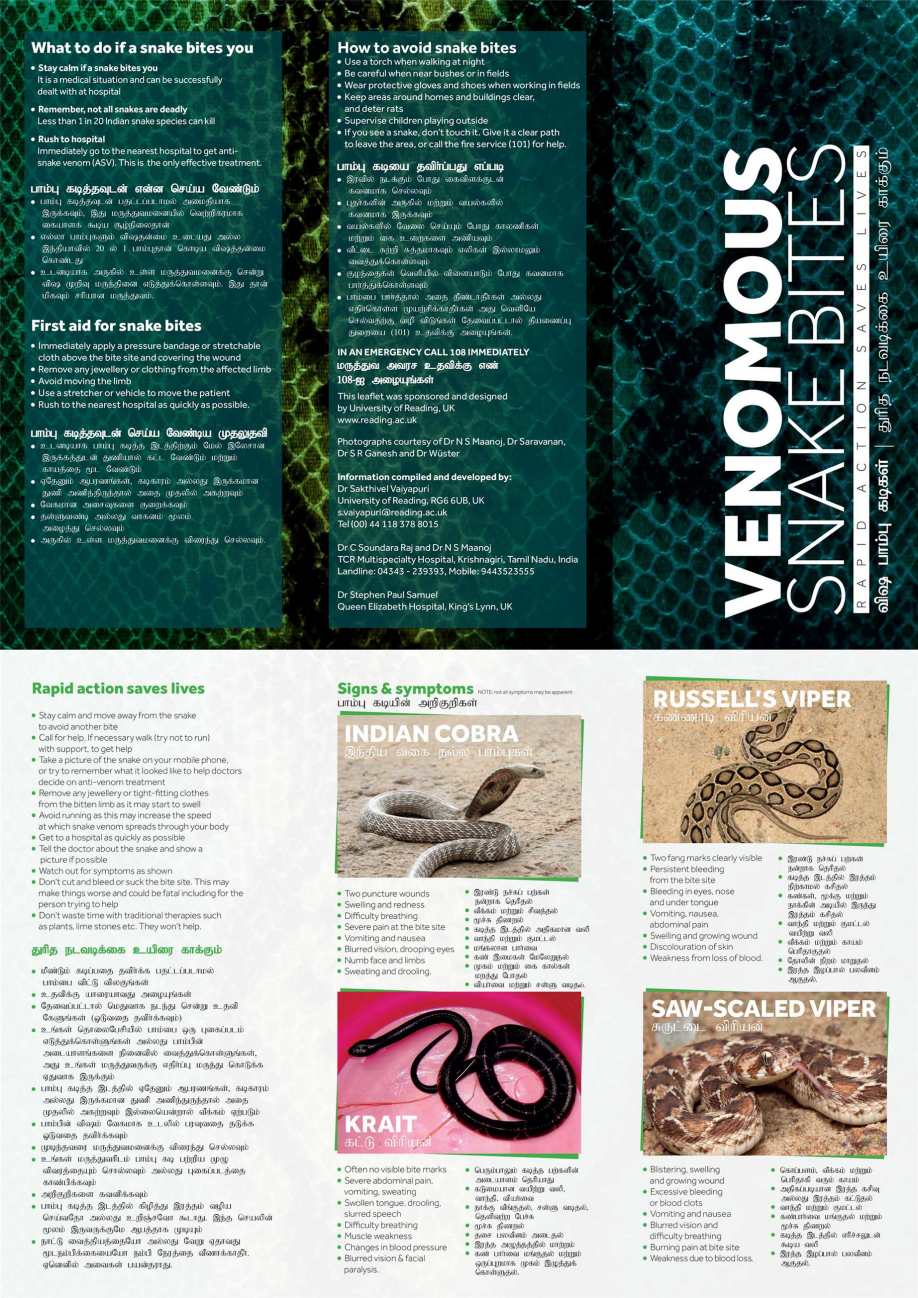
SBE information leaflet developed and used in this study.

**Fig 2:**
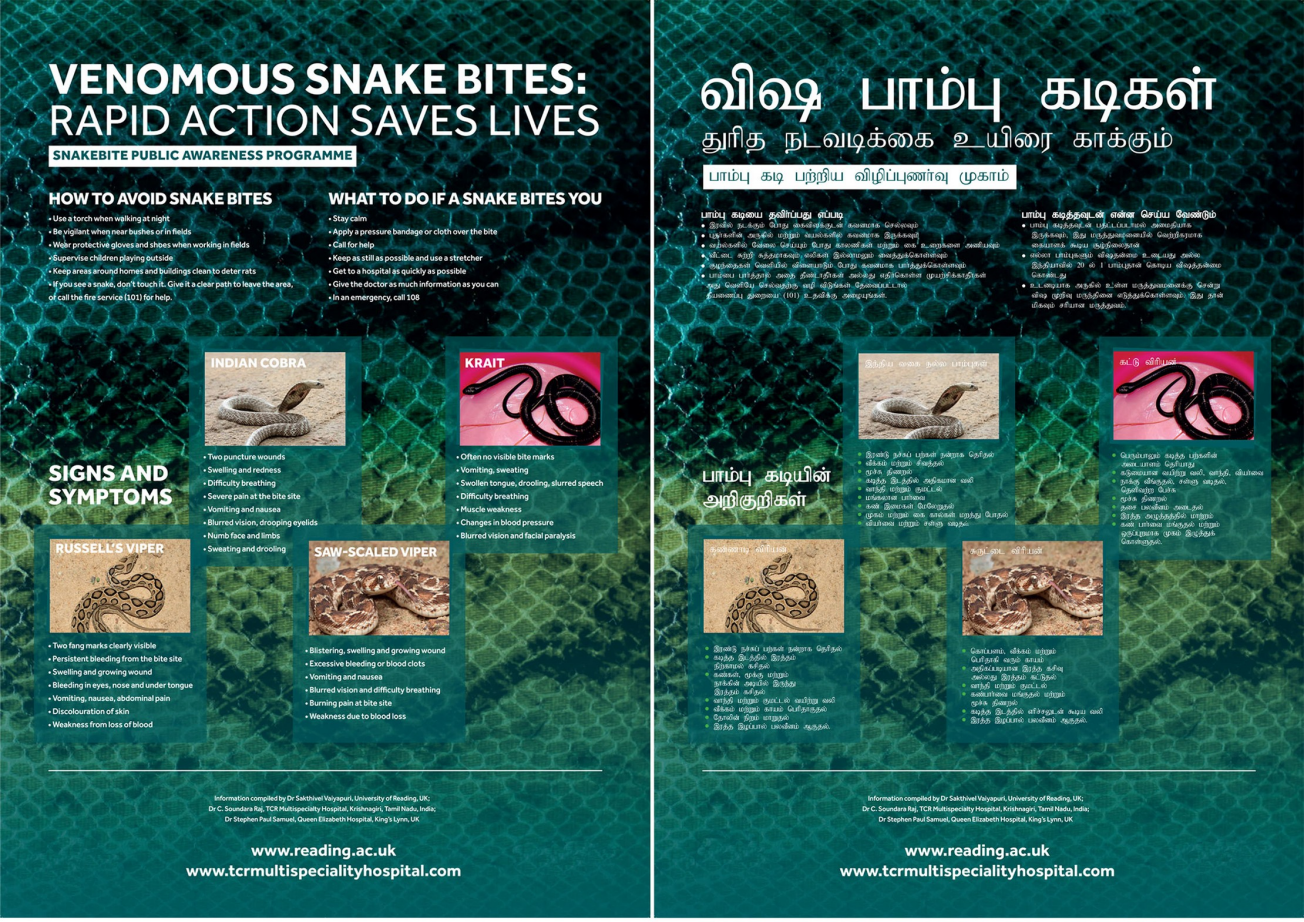
SBE information posters developed and used in this study.

A leaflet (A4 size) containing all of the campaign’s key messages (in Tamil and English) was developed by the authors in conjunction with the Press Office and Research Communications & Engagement team at the University of Reading to reinforce the information provided in our video (Fig 1). Similarly, large (A0 size) accompanying posters were developed with condensed information both in Tamil and English (Fig 2) for display in public places. The leaflet and poster contents were verified and reviewed by researchers in SBE, clinicians (other than the authors) and public engagement professionals both in the UK and India. The leaflet and posters were piloted with a small number (50) of students and members of the public in Tamil Nadu to determine the easy understanding of information provided in Tamil. Following successful review and further modifications, 200,000 leaflets and 50 posters were printed (in colour) and used during this campaign.

### SBE awareness assemblies

Engaging with school and college students is an established method for disseminating health information to a wider population [18]. Hence, between January and December 2019, SBE awareness assemblies were organised in around 140 educational institutions including primary, secondary and higher secondary schools, colleges and universities across Tamil Nadu with audience numbers ranging from 200 to 400 in each institution. This was only a small sample compared to the total number of schools (around 58,000), colleges (around 3,000) and universities (around 60) in Tamil Nadu. However, it enabled us to test the approaches and reach a wide range of students studying diverse programmes in different institutions across the state. Students of different age groups participated in these activities; e.g. 5–10 year-old children in primary schools, 11–15 year-old students in secondary schools, 16–18 year-old students in higher secondary schools, and 18 or above in colleges/universities. Each institution was approached by one of the authors or the wider group of team members (comprising selected staff from TCR Multispeciality Hospital, some NGOs and educational institutions) at least three days in advance to arrange the visit on a specific time and day. All the prior arrangements (e.g. setting the assembly hall, arranging students and necessary facilities such as access to electricity) were performed by the institutions and our team organised the audio-visual tools, displayed posters and distributed the leaflets.

In each location, the assembly lasted approximately 90 minutes, which included a brief introduction, showing the video documentary, and then a question and answer (Q&A) session with scientists and clinicians (mainly S.V, S.C, and S.P.S, but in some locations snake rescuers and staff from our NGO partners were also involved). At the end of each session, our leaflets were distributed to all participants. Advice was provided for each student to disseminate the knowledge that they had gained among their families, friends and neighbours. Specific emphasis was put on kraits as these are nocturnal snakes whose bites are initially asymptomatic and painless; hence many victims die or suffer paralysis without realising the cause.

Additionally, in 50 specific locations, short (around 30 minutes) workshops for a small cohort of students (up to 50 who were randomly selected by the institutions) were organised. During these sessions, the students were asked to identify commonly found snakes in their regions from pictures (e.g. ‘Big Four’ snakes, Indian rat snake, Indian python and Indian wolf snake) and different features (e.g. body scales and type of eyes) of venomous/non-venomous snakes and SBE (e.g. symptoms of SBE such as fang marks, bleeding from bite site and ptosis). In a selection of 20 participating institutions, poster competitions (prizes were announced for 3 best posters) were organised so that students could visually illustrate the knowledge that they had gained from our activities to their families, friends and relatives.

The same assembly approach was used in a total of around 100 rural villages in Tamil Nadu. Within each village, people gathered in several common locations. In each location, an audience of around 100–300 people were received, depending on the time of the day. If the events were organised in the evening, then the video documentary was played followed by a Q&A session (totalling around 60 minutes). However, during the daytime sessions, only a brief talk and Q&A session was organised (totalling around 30 minutes). Information leaflets were distributed to all attendees and people were advised to keep them safely in their houses for use at any time. Additional leaflets were distributed via hospitals, small clinics, public health centres, pharmacies, supermarkets, police and forest offices even in locations where the team had no established links.

### Support from local and national media

As it was practically impossible to achieve direct engagement with the entire rural population of Tamil Nadu during this study, support was sought from local and national media to further disseminate the campaign’s key messages over the same time period (January-December 2019). Contacts with media outlets were established through phone calls and emails, building on existing links with local journalists. Numerous newspapers (both in Tamil and English), magazines, television and YouTube channels have disseminated the campaign’s key messages (e.g. how to avoid snakes and do’s and don’ts if bitten by a snake). Notably, a popular Tamil television channel, Puthiya Thalaimurai, developed a 25-minute-long documentary by interviewing a scientist (SV) and clinicians (SPS, SC and Dr Ragunanthanan from Madras Medical College, India) [19]. This documentary covered information about SBE in general, its statistics, the Big Four snakes and symptoms of their bites, how to prevent snakebites and do’s and don’ts if bitten by a snake. Similarly, an online news channel, Update News 360, developed a 5-minute-long documentary with some of the campaign’s key messages (e.g. do’s and don’ts if bitten by a snake and how to avoid snakebites) and shared this via their Facebook page [20]. All media outlets decided to disseminate the essential information we provided about SBE due to its significance to members of the public.

### Dedicated campaign Facebook page

A dedicated Facebook page [21] was launched in early March 2019 to support the campaign primarily in Tamil Nadu. This page provided access to all of our materials (information leaflets, posters, short and full video documentaries), updates of our activities in local communities, and highlighted media coverage of the campaign. Two short (3-minute long) videos (both in Tamil and English) which briefly described the campaign’s key messages were developed specifically for this social media page. These were promoted via two Facebook advertisements during April and May 2019 (over two separate three-week periods) by targeting the rural population of Tamil Nadu (by selecting the specific regions to focus in Facebook) to increase visits to the page. This Facebook page continues to provide SBE-related information and updates.

### SBE symposium for healthcare professionals

Since our previous studies have identified that some healthcare professionals (specifically from rural areas) are not fully aware of essential information about SBE [8,12], we organised a one-day international symposium targeted at healthcare professionals (including clinicians, nurses, pharmacists, and regional public health officers) in Tamil Nadu. The topics in the symposium covered various clinical challenges in handling SBE victims; e.g. surgical procedures to treat SBE-induced muscle damage; complications associated with non-venomous snakebites; and novel strategies for the diagnosis and treatment of SBE. Invitations for this symposium were sent out directly to over 600 healthcare professionals across the state and via the Indian Medical Association. The event was also promoted through advertisements and news articles in daily newspapers. Notably, the symposium was accredited by the Tamil Nadu Medical Council with 4 [Continuing medical education (CME)] credits.

### Evaluating the recall of key messages

A pictorial-based questionnaire, supplemented with questions written in Tamil (S1 Text) with correct/incorrect actions following a snakebite was designed and reviewed by researchers, public engagement professionals and a small (50) cohort of students and members of public to assess awareness of the campaign’s key messages. This was distributed to students and members of the public during the activities in both educational institutions and villages. The questionnaire was self-administered (unless people were unable to read/understand whereupon additional support was provided by one of the team members by reading the questions and recording their answers) and completed anonymously (no personal details were collected). In 50 randomly selected institutions (10 primary and 20 secondary/higher secondary schools and 20 colleges/universities), a total of around 2,000 (approximately 40–50 in each location) students were randomly asked to complete the questionnaire before and after our activities. Similarly, in 50 villages, a sample of around 2,000 (approximately 40–50 in each location) responses were aimed from rural people. The questionnaire was repeated after 12 months in 10 randomly selected educational institutions and 10 villages where the activities and assessments were previously carried out. The locations were selected to be representative of different regions of the state and a small number (around 30 in each location) of students and members of the public were surveyed to assess long-term recall of key SBE messages.

### Monitoring of SBE victims

In order to monitor the changes in treatment-seeking behaviour following snakebites as a result of our activities, routine admissions data was collected (over the same time period as the public education campaign) from SBE patients who were admitted to TCR Multispeciality Hospital, Tamil Nadu—a leading referral centre for SBE victims in the north region of Tamil Nadu. This hospital mostly receives SBE victims from the surrounding six districts (Krishnagiri, Dharmapuri, Salem, Tirupathur, Thiruvannamalai and Vellore), and therefore, all the public engagement activities were condensed in these regions from 1^st^ to 19^th^ January 2019. Patients from these districts can reach this hospital within around 2 to 3 hours based on the mode of transport (taxi, motorbikes and public transports). The hospital data were collected from 20^th^ January until 31^st^ December 2019. Following informed consent from the patients or their relatives, basic details such as snakebite episode, time of bite, first aid, traditional and any additional treatment received were collected by an interviewer (who is normally a duty doctor or nurse). Data were also collected from all patients who were suspected to be snakebite victims (venomous/non-venomous) even where the offending species could not be ascertained. The data were analysed anonymously in order to categorise them for different parameters as discussed in the results section. To compare the data before and after our activities, we also analysed TCR Hospital’s SBE patient admissions records from 2018.

#### Statistical analysis

The sample/population size, selection of locations and evaluation of awareness activities were performed in line with the advice provided by an expert statistician (MFB) at the University of Reading. Tests of association were performed using Pearson’s Chi-squared test and Fisher’s Exact Test for count data with simulated p-value (based on 10,000 replicates) when the assumptions of the Chi-squared test failed. All the statistical analyses were performed using R statistical package [22].

## Results

### SBE assemblies increased awareness among students

**Fig 3:**
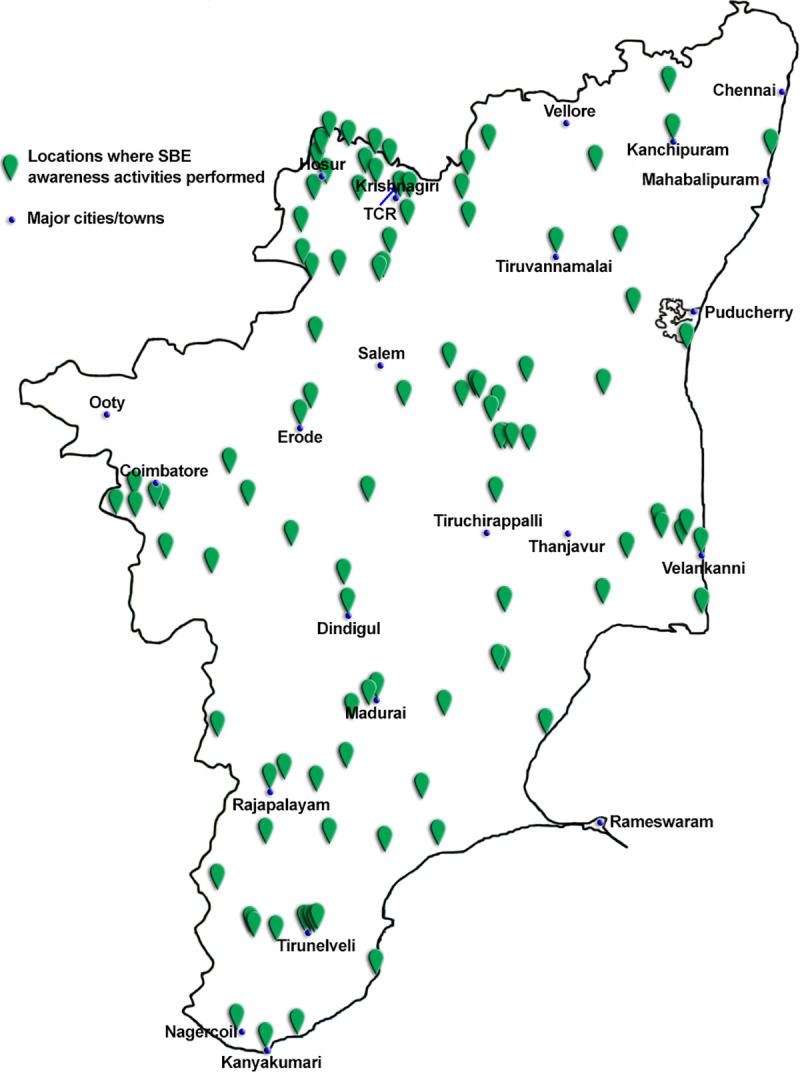
Locations where SBE awareness activities were performed in Tamil Nadu. This map was drawn by the authors based on the Tamil Nadu political map to indicate the locations where SBE awareness activities were performed. This should be used only as a guide as it may not accurately represent the boundaries of the state.

SBE awareness assemblies were conducted in various educational institutions in different locations across rural Tamil Nadu to cover as wide a range as possible (Fig 3). Our network of contacts in the state also expanded during this period, enabling more clinicians, snake rescuers and NGOs to be added to the team. This facilitated visits to institutions across a dispersed area, enabling the team to reach over 50,000 students within a year.

**Fig 4:**
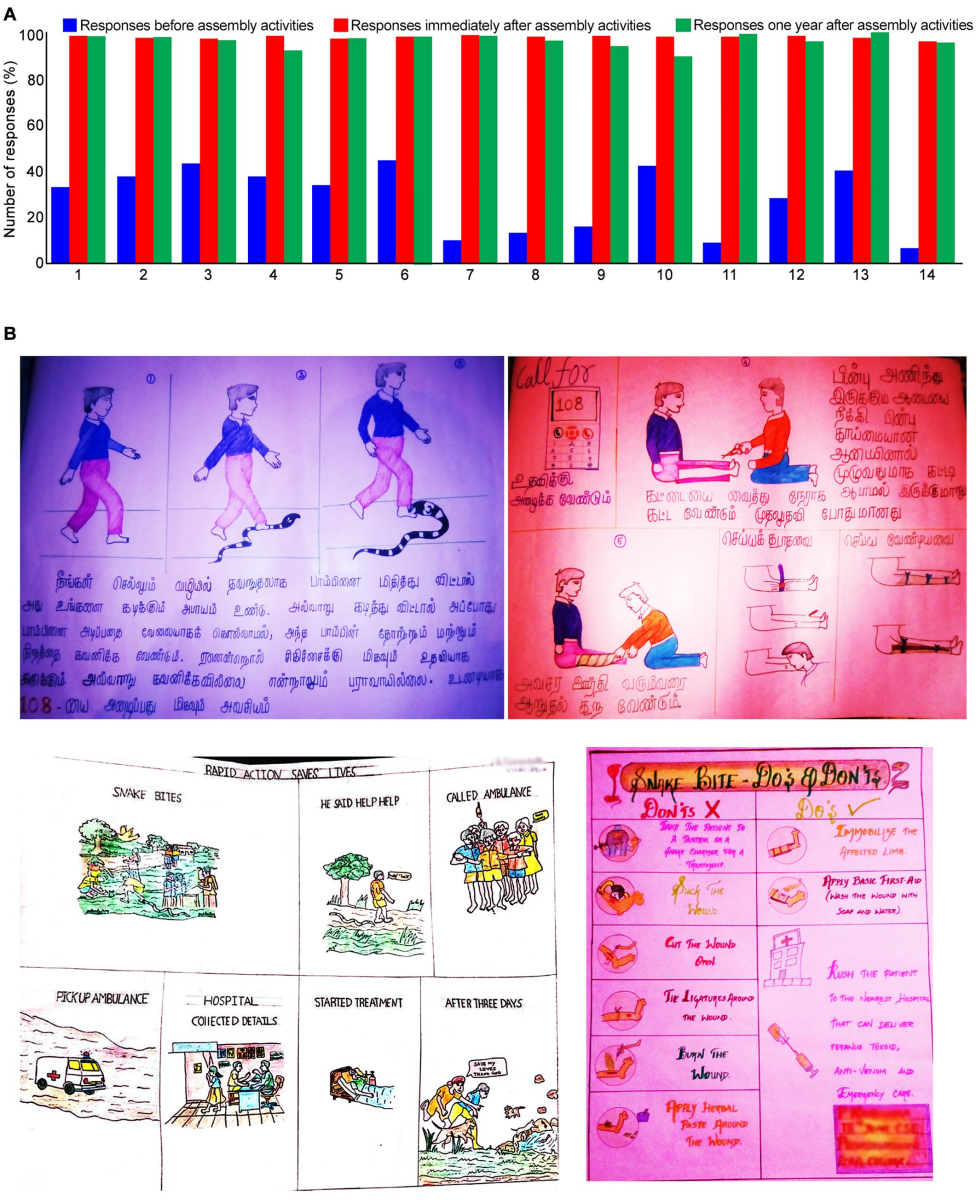
Impact of SBE awareness activities among students in schools and colleges. **A**, student responses to the study questionnaire before (blue) and after (red) our SBE activities (n = 2,004). Furthermore, student responses to the study questionnaire after 12 months (n = 303) (green) were recorded. The number of correct answers was calculated as a percentage for easy comparison. The numbers on the X axis indicate the questions used in the questionnaire; 1) stay calm, 2) wear jewels and tight clothing around the bite site, 3) lay the victim on their back, 4) move slowly, 5) place the victim in a recovery position and reduce movements, 6) seek traditional treatments, 7) seek prompt hospital treatment, 8) panic following a bite, 9) attempt to attack or kill the snake, 10) rinse the eyes with clean water, 11) rub the eyes frequently, 12) incise the bite site and suck the blood, 13) tie with a rope or cloth above the bite site, and 14) leave the bite site untouched. **B**, Example of posters that were drawn by students to visually illustrate the knowledge and information that they learned from our activities.

The pre-event questionnaire indicated that all students (n = 2,004) had some basic awareness of snakes and snakebites (Fig 4A). For example, they were aware that not all snakes are venomous and have the potential to kill people. Around 40% of students were aware of some appropriate responses to SBE; e.g. to put victims into the recovery position and to avoid traditional treatments and tight tourniquets. However, our analysis also identified a number of inappropriate responses to snakes and SBE. Nearly 90% of students thought that prompt medical treatment was unnecessary, that panicking is required, and that leaving the bite site undisturbed is incorrect. A significant number (40%) of students also believed that using calcium carbonate and toxic secretions of plants would help to neutralise snake venoms, with a large number (60%) of participants believing that traditional treatments are the best approach to treat all kinds of bites including non-venomous and non-snakebites. At the end of each session students were asked to repeat the study questionnaire. Here, over 90% of students showed good recall across all key messages (Fig 4A).

During our workshops, a small number of students (around 50 in each randomly selected institution) were asked to identify commonly available venomous and non-venomous snakes, and their specific features. As expected, 100% of students correctly identified Indian cobra (*Naja naja*) due to their distinctive ‘hooding’ behaviour. Interestingly, nearly 50% students were confused between the physical characteristics of the Russell’s viper (*Daboia russelii*) and Indian python (*Python molurus*). Similarly, around 60% students were confused between the Indian krait (*Bungarus caeruleus*) and Indian wolf snake (*Lycodon aulicus*). While 40% students knew that Indian rat snakes (*Ptyas mucosa*) are non-venomous, around 60% students thought that rat snake would bite and inject venom using its tail. Students in these workshops were subsequently taught how to distinguish venomous from non-venomous snakes based on specific features such as keeled body scales and the shape and position of the eyes.

In addition, a poster competition was held in selected institutions (20) which enabled students to visually illustrate the knowledge that they had gained about SBE and demonstrate how they would disseminate this information to their friends and relatives. Examples of these posters are shown in Fig 4B. Our analysis of key message inclusion in 50 posters indicated that all the students have emphasised the necessity to seek immediate medical attention following a snakebite. Further discussions with the students who took part indicated that they would be highly likely to disseminate their new knowledge to their families and friends in order to increase awareness among the community. Three best posters (judging criteria being the inclusion of key messages as well as creativity) were awarded with cash prizes (Rs 2,000 for first, Rs 1,500 for second and Rs 1,000 for third prize) in each institution.

Furthermore, 303 questionnaires were collected from students in 10 selected institutions where previous activities had been conducted. These indicated that, 12 months after our activities, over 90% of students were still aware of all the information provided through the awareness assemblies (Fig 4A).

### SBE community activities increased awareness among rural population

**Fig 5:**
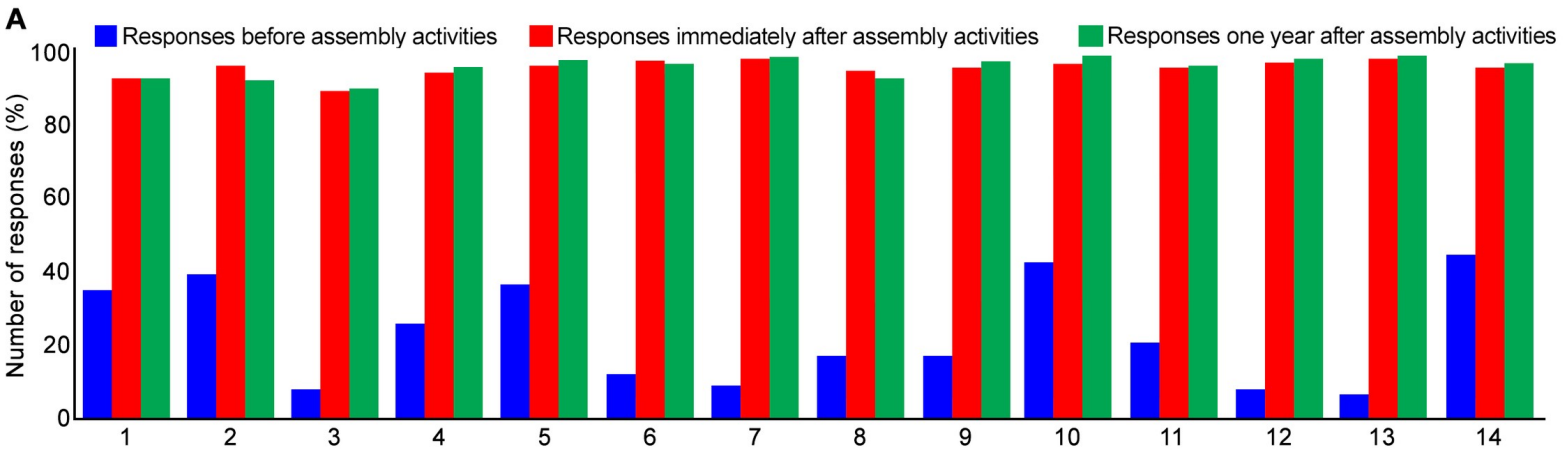
Key message recall among rural people. Responses to the study questionnaire before (blue) and after (red) our SBE activities (n = 2,418). Similarly, responses to the study questionnaire after 12 months (n = 413) (green) were also recorded. The number of correct answers were converted into percentages for easy comparison. The numbers on the X axis indicate the questions used in the questionnaire, as listed in Fig 4.

Events were held in communal locations in around 100 rural villages following a similar format to the school and college assemblies. A total of around 150,000 people were reached over the course of 12 months. Pre-event questionnaire results (n = 2,418) indicated that the vast majority of rural villagers had misconceptions about the appropriate actions to take following a snakebite, with (95%) believing that using one or more tight tourniquets is the best approach to prevent the venom spreading across the body following a bite. Similarly, around 90% people believed that various plant extracts from traditional healers are effective treatments against SBE and other venomous bites (Fig 5). Only 10% of people stated that they would seek immediate medical attention at the nearest hospital. However, post-event questionnaires indicated a 95% recall of the correct first aid measures and actions to avoid following a snakebite (Fig 5). Analysis of key message recall 12 months after our activities in selected villages (where previous activities were conducted) indicated that over 85% of people (n = 413) remembered the information we had provided (Fig 5). In all locations, additional leaflets were distributed through clinics, hospitals, pharmacies and supermarkets and therefore our reach would be higher than we have estimated through attendance at our events.

### Traditional and social media coverage significantly increased the reach of the campaign

Over 60 newspaper articles highlighted our campaign and key messages in 2019, with online and print coverage in local press (e.g. Covaimail, the Kovaiherald, and Kovai Media 24/7 [23]), state-level (e.g. The Hindu Tamil, Dinamalar [24], Dinakaran and Puthiya Thalaimurai Tamil Magazine) and national press (e.g. The Hindu [25–27], The Times of India [28,29], THE WEEK [30], Business Standard [31] and The Federal [32]). Notably, The Hindu has a readership of 6,226,000 in India [33] and 1,880,000 in Tamil Nadu [34] and the Times of India has a readership of 5,650,000 nationwide and 1,150,000 in Tamil Nadu [35]. The Hindu Tamil newspaper has a readership of 2,727,000 in Tamil Nadu [33]. These articles primarily highlighted how common snakebite incidents are in rural India, the methods through which people can avoid snakebites, effective first aid measures, and advised readers to seek immediate hospital treatment following a snakebite.

Similarly, several television channels highlighted the key messages of our campaign during their regular news broadcasts. Notably, a television channel, ‘Puthiya Thalaimurai’ developed a 25-minute long documentary film with our key messages which aired over two weekends at peak times as part of their popular series ‘Pulan Visaranai’ (i.e. Investigation). This documentary is estimated to have been viewed on television by approximately 500,000 people [according to their target rating point (TRP)] and was watched live via YouTube by an additional 2,500 people. Moreover, views of the documentary reached around 14,000 on YouTube within two weeks of broadcasting [19]. Furthermore, Update News 360, an online news channel developed a five-minute-long video documentary, based on our key messages, and disseminated it via their Facebook page to their 425,000 followers [20]. This video reached over 80,000 people within 24 hours and over 330,000 views within one week of being uploaded, with around 7,200 people sharing the video to their own Facebook networks. Similarly, several other social media channels shared our news and video documentaries with their users. The videos, information leaflets and key messages were also shared via WhatsApp to numerous users in Tamil Nadu.

By developing our own dedicated Facebook page for the campaign (https://www.facebook.com/venomoussnakebite/) [21], we have provided a platform both to disseminate our materials, highlight press coverage and for people, mainly across Tamil Nadu, to ask questions and discuss the issues raised. The Facebook adverts that we ran to promote our own short videos were seen by over 2.5 million people (within two separate three-week periods) in rural Tamil Nadu, leading to significant engagements with our Facebook page. Interestingly, we noted that nearly 99% of the adverts’ impressions were via mobile phones. In addition to the high number of visitors drawn by the advertisements the Facebook campaign page has remarkably gained over 11,000 followers (mainly from Tamil Nadu but some from other regions of India). Together, these data demonstrate the significance of using both traditional and social media to increase the reach of the campaign within a short period of time. Due to high number of social media users these days, the exploitation of these platforms for community education is an effective and sustainable approach.

### A symposium about SBE raises awareness among healthcare professionals

Over 250 participants including general physicians, clinicians, nurses, surgeons and other healthcare professionals from Tamil Nadu attended our one-day symposium. In addition to lectures covering the clinical challenges faced by healthcare professionals treating SBE, workshops were held in order to answer the diverse questions from participants and to share current best practice. As part of the symposium evaluation, nearly 95% of participants said that the event was very useful and had changed their views about SBE, and the knowledge that they gained would improve the way in which they handle snakebite victims (although this change in practice was not assessed during this study period). Further written feedback indicated that some participants would like hands on training about appropriate first aid measures for SBE and some suggested having this event over two or three days in the future with additional topics related to SBE covered.

### Community education changed the treatment seeking behaviour of SBE victims

**Fig 6:**
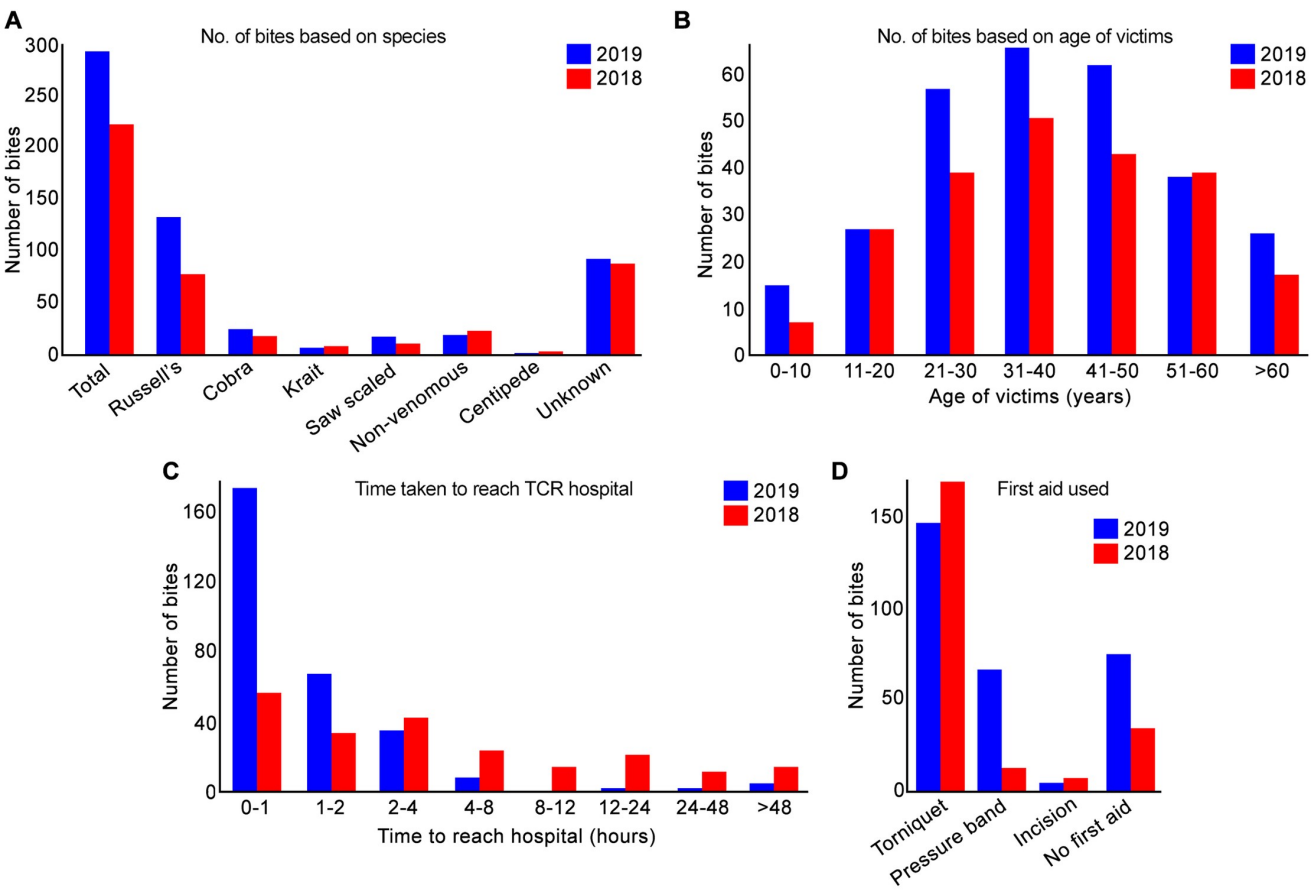
Evaluation of patient data from an SBE referral hospital. **A**, the total number of victims admitted into the study hospital in 2019 (blue) and 2018 (red), and classification of numbers based on the offending snake species. The total number of bite victims admitted in 2019 was significantly (X^2^ = 8.9, df = 1, p = 0.003) higher than those admitted in 2018. The number of bites based on snake species was not (p = 0.098) significantly different between 2018 and 2019 as determined by the Fisher’s Exact Test for Count Data with simulated p-value (based on 10,000 replicates). **B**, age-wise classification of total number of victims admitted in the hospital in 2019 and 2018. The number of victims in age-wise categories was not (X^2^ = 4.6, df = 6, p = 0.593) different in 2019 compared to 2018. **C**, the time taken to reach the hospital following bites. Here, the number of victims admitted within 2 hours in 2019 was significantly increased compared to 2018, while in other categories it was significantly reduced in 2019 (X^2^ = 119.2, df = 7, p < 0.001). **D**, the number of people used (or not used) first aid measures. In 2019, the number of victims received no first aid or applied pressure bandage was significantly increased while the number using a tourniquet significantly reduced compared to 2018 data as calculated using Fisher’s Exact Test for Count Data with simulated p < 0.001, based on 10,000 replicates.

To determine the efficacy of our activities in changing the behaviour of SBE victims and their relatives for seeking prompt hospital treatment, data from 291 patients who were admitted with suspected snakebites during 2019 at TCR Multispeciality Hospital were collected (Fig 6A). In 2018, this hospital received a total of 223 patients, which is significantly lower than in 2019.

Of the 291 admissions in 2019, 132 (45.4%) bites were from Russell’s viper (*Daboia russelii*), 25 (8.6%) from Indian cobra (*Naja naja*), 6 (2%) from Indian krait (*Bungarus caeruleus*) and 17 (5.8%) were from saw-scaled viper (*Echis carinatus*) (Fig 6A). A total of 18 (6.2%) bites were from non-venomous snakes, 1 (0.3%) was from a centipede, and 92 (31.6%) were from unknown bites (where offending species could not be ascertained based on the information/evidence available). Of the 223 admissions in 2018, 77 (34.5%) bites were from Russell’s vipers, 17 (7.6%) from cobras, 7 (3.1%) from kraits and 10 (4.5%) from saw-scaled vipers, 22 (9.9%) from non-venomous snakes, 3 (1.3%) from centipedes and 87 (39%) from unknown species. The number of bites based on the species type in 2019 is not significantly different compared to 2018 data. The identity of offending species was confirmed based on specimens brought to the hospital and descriptions provided by the victims or their relatives in line with the clinical symptoms observed by the clinicians.

In 2019, 162 (55.7%) victims were males and 129 (44.3%) were females. A similar ratio of bite admissions by gender was observed in 2018 with 58.7 to 41.2% male to female respectively. Likewise, the age of SBE patients varied little between 2018 and 2019 (Fig 6B) with the majority of admissions observed in people of working age: 76.6% and 77.2% in 2018 and 2019 respectively were in people aged between 21 and 60.

Significant differences were observed in arrival times at the hospital between the 2018 and 2019 data (Fig 6C) with 276 (95%) arriving in under four hours in 2019, compared to the corresponding figure of 135 (60.5%) in 2018.

In terms of the first aid measures that were reported in admissions data for 2018 and 2019, differences were observed that reflect greater awareness of more effective actions (Fig 6D). This is most notable in the data related to the increased use of pressure bandages (Fig 6D) and the lower uptake of traditional remedies in 2019. Remarkably, 283 (97.3%) (2019) in contrast to 142 (63.7%) in 2018 arrived without seeking traditional treatment, while 8 (2.7%) (2019) and 81 (36.3%) (2018) received some form of traditional treatment such as feeding on some green leaves or herbal powder and applying lime stone or calcium carbonate at the bite site.

These data are further supported by the observation that 173 (59.5%) of those admitted in 2019 reported that they were aware of our campaign, all of whom arrived at the hospital within 4 hours of receiving a bite. None of them sought any traditional treatment, and over 80% had either used a pressure bandage correctly or arrived at the hospital without practicing any first aid. Furthermore, the length of hospital stay for those patients who had arrived within 4 hours of being bitten was less than three days in a large proportion of cases (45%) as they sought prompt medical treatment.

## Discussion

SBE is a disease of poor communities, predominantly affecting the lives of people living in rural areas of developing countries [3,36,37]. Lack of public awareness of appropriate responses to SBE has been identified as a key factor in augmenting SBE-induced deaths and disabilities among such rural groups [8,13]. Data (over 90% in the locations visited) from the pre-questionnaire used in the current study indicated the extent to which rural people believe that traditional treatments are a major solution for SBE. The continued practice of traditional treatments and inappropriate first aid measures leads to delays in obtaining essential treatment, which in itself is a principal cause of deaths and disabilities as well as substantially increasing treatment costs [14]. One-off direct costs associated with SBE treatment can equate to anything from around half a month’s salary to over 12 years’ worth of salary for the average Indian agricultural worker [8]. As many rural people are not usually covered by any medical insurance, such treatment costs can have major long-term socio-economic impacts on survivors and their families [8].

The literature on previous studies from different countries reports on the impact of activities to raise community awareness of SBE in rural areas. For example, a study conducted in Sri Lanka reported the awareness, views and knowledge of SBE among farmers [15]. A smaller study conducted in a rural population of Nepal described the effect of community education and the provision of transport to medical facilities in reducing the incidence of SBE and the number of deaths [38]. A second study in a small rural community of Nepal reported the impact of first aid training on the management of SBE victims [39].

In this study, our primary aim was to dispel myths around snakes and snakebites, and provide clear/simple advice on how to respond to snakebites and, ultimately to positively influence treatment seeking behaviour of victims and their relatives. Throughout our 12-month study, the high level of support that we received from NGOs, members of the public, healthcare professionals and various officials demonstrated the significant public interest in this neglected public health topic. This was also a key factor in enabling us, starting out as a small team of around 15 members and growing to over 100 team members by the end of this period, to reach a large population (>200,000 through direct engagement and >2.8 million via social media) within such a short period of time. Evaluation of our community engagement activities indicated a high level of recall of our key messages by students and members of the public, both at the end of each event (90 and 95% respectively) and after 12 months (85 and 90% respectively). This was in direct contrast to the low level of knowledge of key messages prior to our intervention, suggesting that the approach was effective in its goal to communicate clear advice on appropriate responses to snakebites. Television is an established channel for the dissemination of public health information [40,41]. The governments of India and Tamil Nadu have used this medium to disseminate information about the risk of smoking tobacco, cancer, cardiovascular diseases and, more recently, COVID-19. Using this approach during our study allowed us to reach a large number of people across the state. With viewing figures from one particular documentary exceeding half a million people, and coverage of the campaign in news programmes across several TV channels adding significantly to the reach of our key messages. These figures were augmented yet further via social media, with one video reaching over 330,000 people within a week of being uploaded.

There are an estimated 18 million Facebook users in Tamil Nadu (out of 72 million population), a number that continues to grow. While there are some drawbacks reported in using social media, it can be used effectively for health education due to the huge number of users from the wider community [42]. Interestingly, analysis of our Facebook advertisements showed that 99% of the 2.5 million hits were via mobile phones, which recognises the widespread availability of smart phones and high-speed internet at affordable prices. This enables easy access of social media for many low- and middle-income families. It should be stated however, that Facebook users in India are predominantly men, and our data report that the majority of impressions from our adverts were from male users. This is of concern as women make up around 47% of agricultural labourers in rural India, and they are therefore noticeably not being reached via Facebook. Nevertheless, it is clear that social media played a big role in extending the reach of our campaign, helping to increase awareness of SBE among the rural population of Tamil Nadu.

Continuing medical education (CME) programmes are an effective and vibrant platform by which to train and educate healthcare professionals about emerging diseases and advances in medical technologies. Yet doctors in rural areas have little to no access to such courses. Evaluation of our one-day symposium suggested that similar symposia or training events on SBE would be well received by rural healthcare professionals. This would improve the overall knowledge base among a range of healthcare professions, giving practitioners more confidence in treating SBE victims especially in under-resourced rural medical settings. Currently, clinicians who practice in rural medical settings frequently refer victims to distant tertiary care facilities without providing any initial treatment and thus exacerbating SBE-induced complications. A previous study gauged the awareness of essential first aid measures for SBE among medical students in Nepal [43]. Another study conducted among clinicians practicing in the Chinese military suggested improving their knowledge of SBE first aid protocols by providing appropriate information materials and found that effective training would aid in effective management of SBE [44]. In Australia, several first aid protocols and efficient SBE management protocols for emergency nurses/healthcare professionals have been produced [45] and they will no doubt play a key role in keeping Australia’s SBE mortality at its current, low level. Since healthcare professionals play a critical role in saving lives after SBE, in all countries experiencing high levels of SBE they need to have adequate access to reliable information and appropriate training about SBE, specifically in terms of first aid and standard treatment protocols.

Evaluating the impact of community educational activities on treatment seeking behaviour is a challenging task due to a number of factors, including the time period required to observe a demonstrable change. In order to facilitate such a change as a result of our campaign, we initially focussed our activities in the catchment areas of TCR Multispeciality Hospital during the first three weeks of the campaign, covering as many villages as possible in those regions. The 2019 hospital admissions data subsequently recorded that 60% of the people arriving at this hospital with suspected snakebites were aware of our activities, all of whom had arrived within 4 hours. In fact, comparing all suspected snakebite admissions, a significantly greater number of people arrived at the hospital within 4 hours without seeking any traditional remedies in 2019 compared to 2018. Many of those admitted in 2019 had also correctly applied a pressure bandage as first aid or did not use any first aid method, instead ensuring they reached the hospital promptly, which suggests that the information we have provided to these communities had a positive impact on treatment seeking behaviour. This data suggests that the community education approaches that we have used in this study were effective in changing treatment seeking behaviour of SBE victims or their relatives. Moreover, seeking prompt hospital treatment has reduced the hospital stay in most cases and therefore the treatment cost to the victims and their families. Although a large number of people responded that traditional treatments are effective for SBE during the campaign activities, 97% of people (in 2019) arrived at the hospital without seeking any traditional treatment following our activities. However, it is unclear whether people who did not attend our campaign activities received the key messages via media or social media or students/friends/neighbours. We also cannot rule out the possibilities of numerous other factors such as increased access to transport and unavailability of traditional healers influencing these numbers in 2019.

While the government hospitals and primary health centres provide free treatment for SBE, the majority of people in Tamil Nadu prefer to seek treatment (not only for SBE but for other illnesses too) from private hospitals where they need to pay for treatments. This preference for private hospitals is caused by a range of factors, including prompt, easily accessible and higher quality treatment. Notably, the recently introduced Chief Minister’s *Comprehensive Health Insurance Scheme* in Tamil Nadu [46] provides coverage for people at various levels to obtain treatment from private hospitals. For example, SBE treatment (with antivenom) in wards (Rs 5,400), in high dependency units (HDU) (Rs 8,100) and in intensive care units (ICU) (Rs 10,800), and SBE treatment requiring ventilatory support (up to a maximum of Rs 31,500) is covered under this scheme [46]. Despite government’s initiative, in most cases, this insurance cover may not be sufficient to pay the full treatment costs in private hospitals due to the severity of SBE-induced complications. Hence, the government of Tamil Nadu could consider to improve the treatment-seeking behaviour of people in government hospitals for SBE and/or increase the insurance coverage allowing them to seek prompt treatment in private hospitals at free of charge. This will prevent the significant socio-economic burden on poor agricultural families caused by SBE.

### Limitations of this study

Caution should be applied in drawing conclusions from this study, specifically beyond the sample population. Due to the limited availability of resources, this study was conducted in a single state, Tamil Nadu, which is one of the 29 states (in addition to 7 union territories) in India. Out of 37 million people living in rural Tamil Nadu, using all the approaches, we have directly (through physical activities) and indirectly (via social media) reached an estimated 3 million people, less than 10% of the rural population of this state. However, if these people managed to disseminate the information among their friends, families and communities as requested, this figure could be much higher than this. We have also measured public awareness of SBE before and after our activities within a small cohort of students and rural people. Despite this, the results indicated the effective nature of the activities performed. We also analysed the recall of key messages of participants after only one year and this indicated that they still remember the majority of the information provided. However, this analysis should be continued further to understand their retention of the information beyond one year. Moreover, although we had been informed by some of the study participants that either themselves or their relatives were bitten by snakes, we did not officially collect any details about snakebite incidents among the students and rural people who attended our activities as this was beyond the scope of this current study. We also did not ask the study participants if they had a chance to apply the knowledge that they gained during any snakebite incidents over the one-year period. These details will provide further evidence on the impact of our activities and actual changes in treatment seeking behaviour for SBE in the community. Additional studies and analysis among other rural communities in India and other countries would also provide a more accurate picture of the impact of this community education approach.

Similarly, most of the healthcare professionals highlighted that the knowledge and skills that they gained from the symposium will help to improve their practice. Hence, further training and educational sessions for rural healthcare professionals will change the views and practice of these essential frontline workers in fighting SBE. However, it is difficult to confirm this change in their practice without further follow up studies. We also analysed the snakebite admissions data in a private hospital for one year. However, we were unable to collect similar data in other SBE referral hospitals in the state where we also carried out our activities. Although, we noticed a significant change in some aspects of treatment seeking behaviour of victims and/or their relatives, there might be various other factors (e.g. increased access to transport facilities, income, unavailability of traditional healers and SBE awareness from other sources) influencing these data. A number of limitations learned from this study could be rectified by teams managing similar projects with additional resources and longer study periods.

### Costs and challenges

The total of cost of this study over one year was around £25,000 GBP. This cost includes printing leaflets (around £5,000 GBP), printing posters (£250 GBP), conducting a symposium (around £5,000 GBP), audio-visual tools, incentives for media personnel, Facebook advertisements (£500 GBP), internal travel, fuel charges for the authors’ own vehicles, salaries for staff who did the data collection in the hospital (around £2,500 GBP), cash prizes for poster competition, stationaries and subsistence for all the team members during activities.

This exhaustive programme has also encountered some challenges. For example, time was a critical factor during these activities as we aimed to reach a significant number of locations within a short period. In areas where we did not have any contacts, it was difficult to arrange activities without directly approaching the institutions and villagers which also took a considerable amount of time. Moreover, in some areas, the Q&A session took much longer than anticipated because of the number of questions that people asked. Parallel data collection and monitoring was also a challenging task during these activities. In a very few areas, people were not keen for our activities as they thought that they have sufficient information and nothing more to learn about SBE. Similarly, in some private hospitals, they were not happy to distribute our leaflets due to another private hospital’s name being present on the leaflets. Without our efficient team members, this programme would not have been possible to achieve within a year, and therefore manpower is another critical factor in designing and executing this type of study.

## Conclusions

The multidimensional activities reported in this study can be used as a framework to educate more people in rural areas of Tamil Nadu, and more widely in India and other developing countries where SBE is a major concern. Repeated and continuing activities (either directly through physical activities or indirectly via traditional and social media) in collaboration with local NGOs, healthcare professionals and relevant government officials will significantly enhance awareness about the dangers of SBE and essential first aid and protective measures. These in turn may significantly reduce SBE-induced deaths and disabilities in rural regions and enhance the livelihood of these vulnerable people for SBE. Although we cannot guarantee that everyone who becomes aware of the correct actions following snakebites will change their treatment seeking behaviour, at least we should provide essential knowledge which might demonstrate changes in the community gradually. Prevention is better than cure—therefore a small amount of government spending on this and funding from NGOs to improve awareness about snakes and SBE could significantly reduce the treatment costs if people were to seek prompt treatment without practicing inappropriate first aid or ineffective traditional treatments. However, we cannot rule out the financial constraints/challenges in developing countries, and their health priorities on other life-threatening diseases. Scientific communities should also join together in order to improve public awareness about SBE among the rural populations in relevant countries.

## Supporting information

S1 Text The pictorial questionnaire that was used to evaluate the knowledge of people on correct measures to take following a snakebite. This questionnaire was used before and after our campaign activities to estimate the knowledge of participants and evaluate the impact of awareness programme. The specific actions are written in Tamil, and English translation is as provided in Fig 4 legend (from 1–14).
(PDF)
