# Supplementary material for: Venomous snakebites: Rapid action saves lives—A multifaceted community education programme increases awareness about snakes and snakebites among the rural population of Tamil Nadu, India
Source: PLoS Negl Trop Dis. 2020 Dec 31;14(12):e0008911. doi: 10.1371/journal.pntd.0008911 (PMC7774832; doi:10.1371/journal.pntd.0008911)
Supplement: S1 Text — This questionnaire was used before and after our campaign activities to estimate the knowledge of participants and evaluate the impact of awareness programme. The specific actions are written in Tamil, and English translation is as provided in Fig 4 legend (from 1–14). (PDF) [file pntd.0008911.s001.pdf]

பாம்பு கடிக்கான முதலுதவி செயல்கள்

பின்வரும் படங்களில் சரி(✓) அல்லது தவறு(×) என தேர்ந்தெடுத்து நிரப்புக

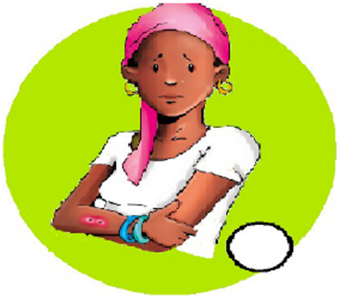

அமைதி காத்தல்

1

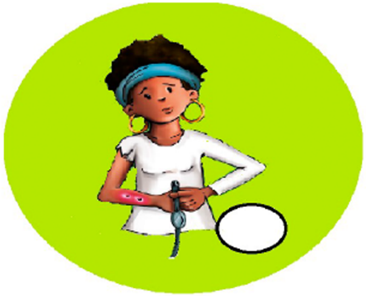

பாதிக்கப்பட்ட பகுதியை சுற்றி இறுக்கமான பொருட்களை அணிதல்

2

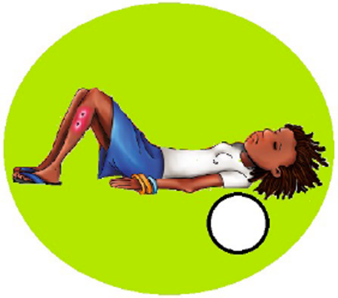

நோயாளியை முதுகுபுறமாக அல்லது பின்புறமாக படுக்க வைத்தல்

3

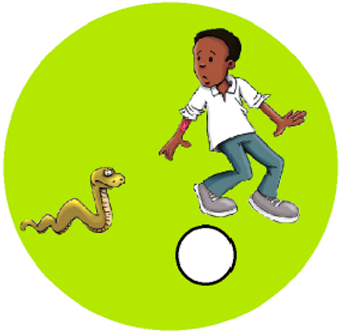

மெதுவாக நகர்தல்

4

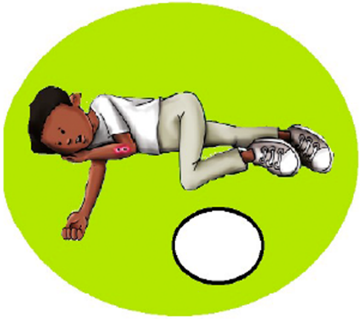

காயபட்ட பகுதியை அசைக்காமல் படுக்க வைத்தல்

5

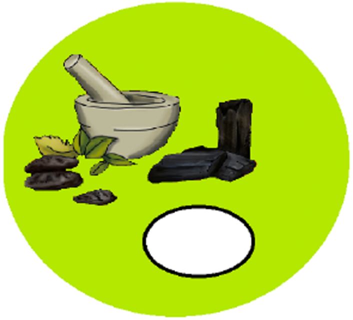

பாரம்பரிய சிகிச்சை முறைகளை பயன்படுத்துதல்

6

Graphics, courtesy of ‘Health Action International’

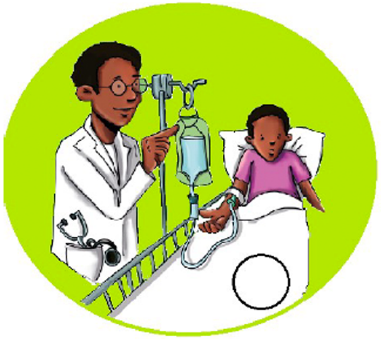

மருத்துவ சிகிச்சை அளித்தல்

7

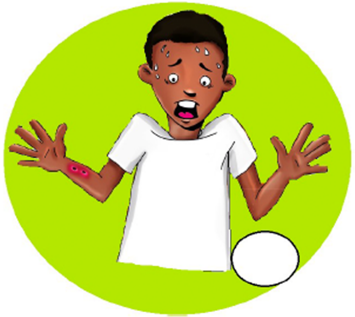

பதற்றம் அடைதல்

8

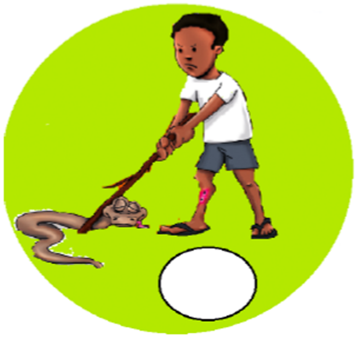

பாம்பைத் தாக்குதல் மற்றும் கொல்லுதல்

9

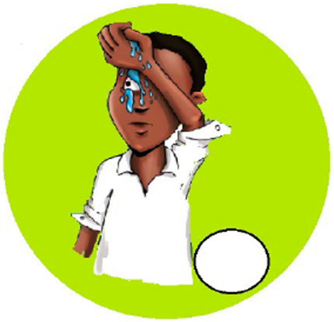

கண்களை ஓடும் நீரில் கழுவுதல்

10

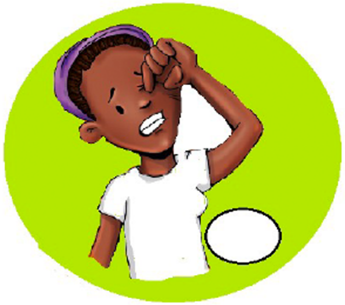

கண்களைத் தேய்த்தல்

11

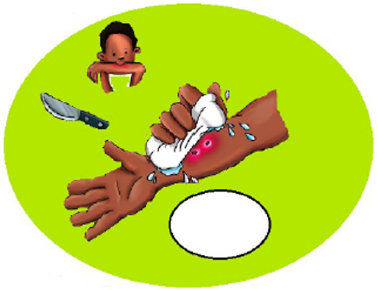

காயப் பட்ட பகுதியை கழுவுதல் மற்றும் உறிஞ்சுதல்

12

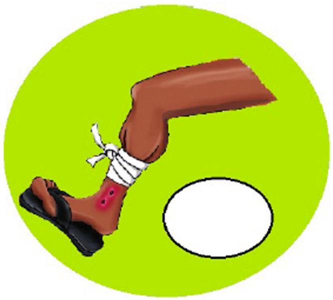

இரத்த ஓட்டத்தை நிறுத்த பாதிக்கப்பட்ட பகுதியை கட்டுதல்

13

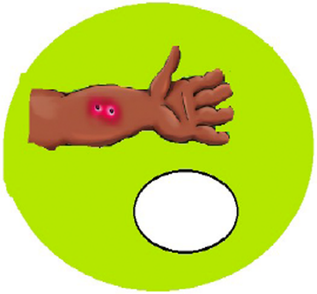

காய பகுதியை தொடாமல் இருத்தல்

14

Graphics, courtesy of ‘Health Action International’
